# Antibacterial activity and mechanism of action of auranofin against multi-drug resistant bacterial pathogens

**DOI:** 10.1038/srep22571

**Published:** 2016-03-03

**Authors:** Shankar Thangamani, Haroon Mohammad, Mostafa F. N. Abushahba, Tiago J. P. Sobreira, Victoria E. Hedrick, Lake N. Paul, Mohamed N. Seleem

**Affiliations:** 1 Department of Comparative Pathobiology, Purdue University College of Veterinary Medicine, West Lafayette, IN, USA; 2 Faculty of Veterinary Medicine, Assiut University, Assiut, Egypt; 3 Bindley Bioscience Center, Purdue University, West Lafayette, IN, USA

## Abstract

Traditional methods employed to discover new antibiotics are both a time-consuming and financially-taxing venture. This has led researchers to mine existing libraries of clinical molecules in order to repurpose old drugs for new applications (as antimicrobials). Such an effort led to the discovery of auranofin, a drug initially approved as an anti-rheumatic agent, which also possesses potent antibacterial activity in a clinically achievable range. The present study demonstrates auranofin’s antibacterial activity is a complex process that involves inhibition of multiple biosynthetic pathways including cell wall, DNA, and bacterial protein synthesis. We also confirmed that the lack of activity of auranofin observed against Gram-negative bacteria is due to the permeability barrier conferred by the outer membrane. Auranofin’s ability to suppress bacterial protein synthesis leads to significant reduction in the production of key methicillin-resistant *Staphylococcus aureus* (MRSA) toxins. Additionally, auranofin is capable of eradicating intracellular MRSA present inside infected macrophage cells. Furthermore, auranofin is efficacious in a mouse model of MRSA systemic infection and significantly reduces the bacterial load in murine organs including the spleen and liver. Collectively, this study provides valuable evidence that auranofin has significant promise to be repurposed as a novel antibacterial for treatment of invasive bacterial infections.

Bacterial resistance to antibiotics is a significant public health challenge, as infections caused by antibiotic-resistant bacteria claim the lives of nearly 23,000 people each year in the United States alone^1^. A single pathogen, methicillin-resistant *Staphylococcus aureus* (MRSA), is responsible for nearly half of these fatalities. MRSA has been linked to invasive diseases including pneumonia^2^ and sepsis^3^, that affect a diverse population of patients including individuals with a compromised immune system^4^ such as young children^5^. While a powerful arsenal of antibiotics was once capable of treating *S. aureus*-based infections, clinical isolates of MRSA have emerged to numerous antibiotics, including agents of last resort such as vancomycin^6^ and linezolid^7^.

Most current antibiotics were discovered via the time-consuming and financially taxing process of *de novo* synthesis and screening of chemical compounds^8^. An alternative approach to unearthing new antibacterials that is garnering more recent attention is screening libraries of approved drugs (or drugs that made it to clinical trials but ultimately failed to receive regulatory approval) in order to identify candidates that can be repurposed as antimicrobials^8^. Recently, we assembled and screened 50% of the commercially available drugs (~2,200 drugs) and small molecules tested in human clinical trials^9^,^10^ (727-NIH Clinical Collections 1 and 2, 1,600-Pharmakon from Microsource, Approved Oncology Drugs Set-NIH, and few small libraries) and identified three drugs that exhibited potent antibacterial activity at a dose that is clinically achievable. One of these drugs, auranofin, is capable of inhibiting growth of clinically-pertinent isolates of MRSA at submicrogram/mL concentrations *in vitro*. Auranofin is an oral gold-containing drug initially approved by the U.S. Food and Drug Administration (FDA) for treatment of rheumatoid arthritis. In a study by Debnath *et al.* auranofin was found to exhibit potent anti-parasitic activity against *Entamoeba histolytica* providing evidence that this drug could be repurposed as an antimicrobial agent^11^. More recent studies have discovered this drug also possesses potent antibacterial activity including against important pathogens such as MRSA^11^,^12^,^13^,^14^,^15^.

Building upon this seminal work, the goals of the present study were to further investigate the antibacterial mechanism of action of auranofin and to examine potential applications of auranofin as an antibacterial agent for systemic MRSA infections. We have identified that auranofin appears to target multiple biosynthetic pathways in *S. aureus*, including inhibition of cell wall, DNA, and protein synthesis; this latter property permits auranofin to mitigate specific virulence factors including reducing the production of key toxins such as α-hemolysin and Panton-Valentine leukocidin, a fact previously unknown. Auranofin is less effective against Gram-negative pathogens in large part due to the presence of the outer membrane in these pathogens. Furthermore, *in vivo* studies demonstrate that auranofin is capable of treating invasive MRSA infections, thereby expanding the potential therapeutic applications of this drug for use as a novel antibacterial agent. The findings presented in this study provide strong evidence that auranofin can be repurposed as a novel antibacterial agent for treatment of invasive MRSA infections in humans.

## Results

### Auranofin is a potent inhibitor of multidrug-resistant Gram-positive bacteria

The antimicrobial activity of auranofin was assessed against a panel of clinical isolates of multidrug-resistant Gram-positive pathogens using the broth microdilution method (Table 1). Auranofin exhibited potent bactericidal activity against all tested bacteria including strains that are resistant to conventional antimicrobials such as methicillin and vancomycin. The minimum inhibitory concentration (MIC) of auranofin, required to inhibit growth of different MRSA strains, were found to be in the range of 0.0625 to 0.125 μg/ml (Table 1 and Supplementary Figure 1). The antibacterial activity of auranofin against MRSA is superior (16-fold lower MIC for auranofin) to several commercial antibiotics including vancomycin (MIC of 1 μg/ml) and linezolid (MIC ranged from 2–4 μg/ml); the MIC values determined for auranofin against MRSA correlate with MIC values reported in previous published studies^12^,^14^. Auranofin retained its antibacterial activity against an array of MRSA strains exhibiting resistance to numerous antibiotic classes including glycopeptides, oxazolidones, tetracycline, β-lactams, macrolides, and aminoglycosides; these results indicate that cross-resistance between these antibiotics and auranofin is unlikely to occur. The bactericidal activity of auranofin was confirmed via a standard time-kill assay (Supplementary Figure 2); auranofin, at 5 × MIC, exhibited slow bactericidal activity (similar to vancomycin), completely eliminating MRSA USA300 cells within 48 hours. Vancomycin required 24 hours to achieve the same effect, which is in agreement with previously published reports^16^. In addition to possessing anti-MRSA activity, auranofin also exhibited potent antibacterial activity against vancomycin-sensitive enterococcus and vancomycin-resistant enterococcus (VRE), *Streptococcus pneumoniae* and *Streptococcus agalactiae* with MIC values ranging from 0.0015 to 0.25 μg/ml.

### The outer membrane in Gram-negative bacteria negates auranofin’s antibacterial activity

Confirmation of auranofin’s potent antibacterial activity against multiple Gram-positive pathogens led us to analyze if auranofin exhibits broad-spectrum antibacterial activity by also inhibiting growth of important Gram-negative pathogens. Interestingly, auranofin alone did not show activity against Gram-negative bacteria which is in agreement with previous reports^12^,^13^,^14^. We sought to investigate if the presence of the outer membrane (OM) in Gram-negative bacteria contributed to the lack of antibacterial activity observed, by preventing auranofin from gaining entry into the bacterial cell (as has been observed with conventional antimicrobials such as erythromycin and fusidic acid)^17^,^18^. The inclusion of the permeabilizing agent polymixin B nonapeptide (PMBN), at a sub inhibitory concentration, in the culture broth resulted in auranofin exhibiting potent activity against all tested strains of Gram-negative pathogens including *Pseudomonas aeruginosa, Escherichia coli*, metallo-β-lactamase (NDM-1) and carbapenemase-resistant (KPC) *Klebsiella pneumoniae, Salmonella Typhimurium* and extremely drug-resistant (XDR) *Acinetobacter baumannii* with MICs ranging from 0.125 to 1 μg/ml (Table 2). In addition to this, a four-fold decrease in auranofin’s MIC (from 32 to 8 μg/ml) was observed when the efflux pump AcrAB was deleted in *E. coli.* AcrAB has been shown to contribute to the antibiotic-resistant phenotype in multiple strains of *E. coli* and has been implicated in *E. coli* resistance to numerous antibiotics including ampicillin, rifampicin, and chloramphenicol^19^. Thus, in addition to the physical barrier imposed by the Gram-negative OM, the ability of auranofin to gain entry into Gram-negative bacteria to exhibit its antibacterial activity may be impeded by the presence of efflux pumps (such as AcrAB).

### Auranofin inhibits multiple biosynthetic pathways in *S. aureus*

**Figure 1:**
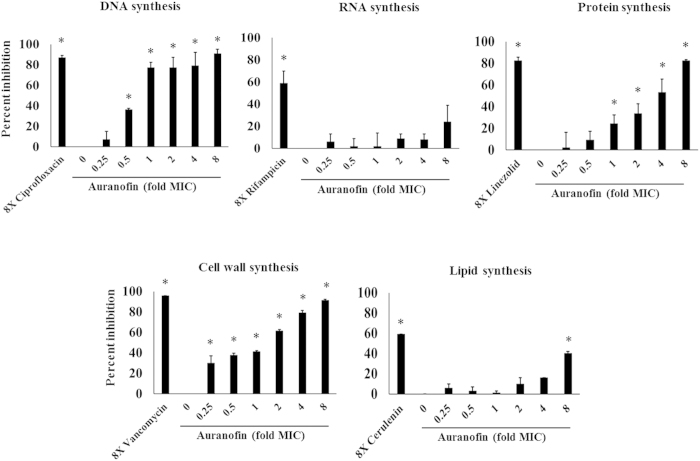
Antibacterial mechanism of action of auranofin examined via the macromolecular synthesis assay. Incorporation of radiolabeled precursors of DNA, RNA, protein, cell wall and lipid synthesis ([3H] thymidine, [3H] uridine, [3H] leucine, [14C] N-acetylglucosamine and [3H] glycerol, respectively) were quantified in *S. aureus* ATCC 29213 after treatment with 1 × and 8 × MIC of auranofin, and 8 × MIC of control antibiotics. Results are expressed as percent inhibition of each pathway based on the incorporation of radiolabeled precursors. Statistical analyses were done using the two-tailed Student’s ‘t’ test. *P* values of (* ≤ 0.05) are considered as significant. Detailed “P” values are listed below. DNA synthesis: control vs ciprofloxacin (8×):0.003, control vs auranofin (0.5×):0.0025, control vs auranofin (1×):0.0005, control vs auranofin (2×):0.0004, control vs auranofin (4×):0.0011, control vs auranofin (8×):0.0003. RNA synthesis: control vs rifampicin (8×):0.0006. Protein synthesis: control vs linezolid (8×):0.0001, control vs auranofin (1×):0.0033, control vs auranofin (2×):0.0048, control vs auranofin (4×):0.0032, control vs auranofin (8×):0.0002. Cell wall synthesis: control vs vancomycin (8×):0.0001, control vs auranofin (0.25×):0.0027, control vs auranofin (0.5×):0.0018, control vs auranofin (1×):0.0005, control vs auranofin (2×):0.0028, control vs auranofin (4×):0.0013, control vs auranofin (8×):0.0003. Lipid synthesis: control vs cerulenin (8×):0.0001, control vs auranofin (8×):0.0003.

After confirming auranofin possesses potent antibacterial activity *in vitro*, particularly against drug-resistant strains of *S. aureus*, we next moved to determine the antibacterial mechanism of action of auranofin. A macromolecular synthesis assay was employed to initially investigate auranofin’s antibacterial mechanism of action. The effect of auranofin on the incorporation of radiolabeled precursors into five major biosynthetic pathways of *S. aureus* was assessed. This assay revealed a clear dose-dependent inhibition of three pathways, indicating that auranofin might possess multiple targets (Fig. 1). Auranofin, at a sub-inhibitory concentration, significantly inhibited cell wall and DNA synthesis. When tested at its MIC, auranofin was found to also inhibit protein synthesis. At higher concentrations (8 × MIC auranofin), partial inhibition of lipid synthesis was also observed. However, auranofin did not significantly inhibit RNA synthesis at any of the tested concentrations. The results from the macromolecular synthesis assay suggest that auranofin possesses a complex mode of action that involves inhibition of multiple biosynthetic pathways including cell wall, DNA, and protein synthesis.

Primary disruption of DNA synthesis in the macromolecular synthesis assay is often associated with DNA intercalators. However, when auranofin was examined for evidence of DNA intercalation, no effect on DNA migration was observed in relation to the untreated control. Unlike doxorubicin, auranofin, even at a concentration (1mg/ml) that is 8000-fold higher than the average MIC against MRSA, shows no evidence of a shift in plasmid DNA (Supplementary Figure 3). These data suggest that the disruption of DNA synthesis by auranofin is not due to intercalation with DNA.

### Auranofin treatment in *S. aureus* leads to downregulation of proteins in five major biosynthetic pathways

**Figure 2:**
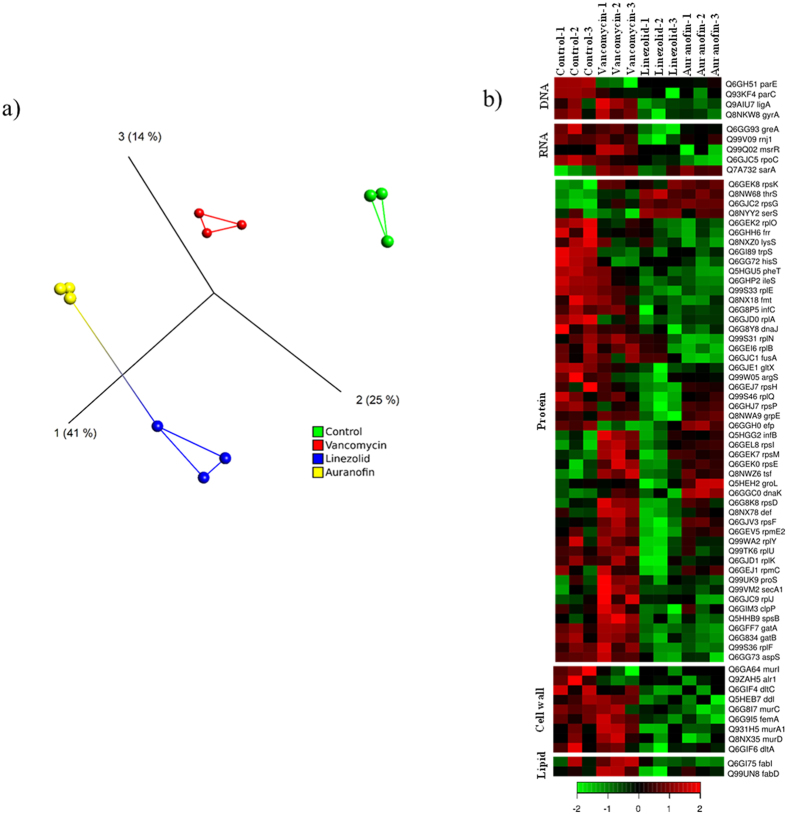
Auranofin treatment in *S. aureus* leads to downregulation of proteins in five major biosynthetic pathways. **(a)** The PCA analysis shown for auranofin, vancomycin, linezolid and control proteins quantified by proteomic analysis. The plot depicts the variance inside each group and the protein expression pattern of drug treated and control groups. **(b)** Heat map generated comparing auranofin-, vancomycin- and linezolid-treated cells to untreated control *S. aureus* cells is shown. Triplicate samples were used for each group. One-way analysis of variance (ANOVA) was used for statistical analysis and the proteins that were significantly differentially (*P* ≤ 0.05) expressed were mapped. Red color indicates significantly increased ratios and green color represents significantly decreased ratios.

Proteomic profiling is a powerful tool that can be employed to investigate the response of bacteria to antibacterial compounds and assess the impact of such compounds on different cellular pathways^20^,^21^,^22^. Therefore, the alteration in the *S. aureus* proteome caused by auranofin was investigated and compared with linezolid and vancomycin in relation to an untreated control group. The proteomic analysis identified 530 proteins in all samples and found 222 of these proteins showed significant differential expression (*P* ≤ 0.05). The PCA analysis demonstrated that the variance inside each group is very low with distinct classifications and the protein expression pattern of the auranofin-treated group resembles that of the linezolid-treated group more so than either the control or vancomycin-treated groups (Fig. 2a).

The proteins were separated into five groups based on molecular function (DNA, RNA, protein synthesis, cell wall and lipid synthesis) (Fig. 2b). Similar to the protein synthesis inhibitor linezolid, treatment with auranofin leads to the down regulation of most of the proteins involved in all five major biosynthetic pathways. The average fold changes (log_2_) of proteins between auranofin and the control group involved in each pathway was: −0.76 (DNA), −0.37 (RNA), −0.26 (protein), −0.76 (cell wall) and −0.18 (lipid). In the presence of auranofin, approximately 55% of the proteins were significantly differentially expressed as compared to the control group (*P* ≤ 0.05). Of the 222 proteins that showed significant differential expression, only 20% of these proteins were upregulated in the auranofin-treated group compared to 40% of proteins that were upregulated in the control group (*P* ≤ 0.05). These results suggest that auranofin treatment leads to significant down regulation of most of the proteins involved in all five major biosynthetic pathways, which contributes to the bactericidal effect of auranofin against *S. aureus*.

### Thioredoxin reductase is not the sole target for auranofin in *S. aureus*

**Figure 3:**
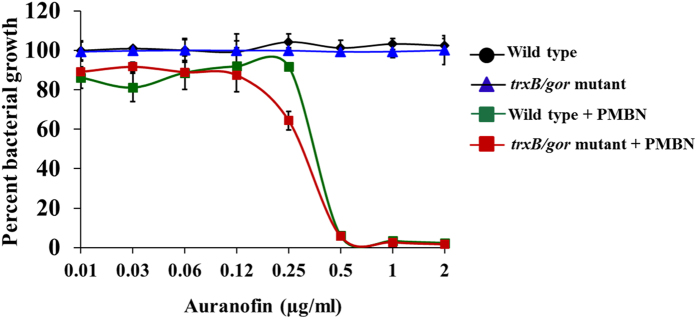
Growth curve of novablue (DE3)-K12 wild-type and *trxB/gor* Origami-2 double mutant *E. coli* strains in the presence of auranofin. *E. coli* strains were incubated with indicated concentrations of auranofin in the presence and absence of PMBN (10 μg/ml) and the growth was measured using a spectrophotometer.

A recent investigation of auranofin as an antibacterial agent^13^ reported that auranofin exerts its bactericidal activity by targeting thiol-redox homeostasis through direct inhibition of the thioredoxin reductase enzyme. The authors postulate that the glutathione system present in certain species of Gram-negative (and Gram-positive) bacteria limits their susceptibility to auranofin (as this system is functionally similar to the thioredoxin system and can maintain redox homeostasis inside the bacterial cell when the thioredoxin reductase enzyme is inhibited). This led the authors to conclude that auranofin’s primary antibacterial mechanism is through inhibition of thioredoxin reductase. While auranofin has been shown to inhibit thioredoxin reductase both in *S. aureus* and *M. tuberculosis*, we suspect that this enzyme is not the sole antibacterial target of auranofin for the reasons outlined below. First, we have confirmed that the lack of antibacterial activity of auranofin against Gram-negative bacteria (as presented in Table 2) is due to the permeability barrier conferred by the outer membrane (OM) and is not glutathione-mediated. Second, an *E. coli* double mutant strain (Origami-2) containing mutations to both the thioredoxin reductase (*trxB*), the purported target of auranofin, and glutathione reductase (*gor*), responsible for maintaining redox homeostasis in the absence of TrxB, genes exhibited identical antibacterial activity to the wild-type *E. coli* strain (Novablue (DE3)-K12) (MIC = 16 μg/ml) (Table 2). However, there is a greater than 32-fold improvement in antibacterial activity of auranofin when combined with a subinhibitory concentration of PBNP (MIC = 0.5 μg/ml) (Table 2). This observation was further validated by assessing the growth of wild-type and the double mutant *E. coli* (Origami-2) strains in the presence of increasing concentrations of auranofin (with or without PBMN) (Fig. 3). Once again, the viability of the Origami-2 double mutant was severely impacted by auranofin in the presence of a subinhibitory concentration of PBMN; however, in the absence of PBMN, the double mutant strain exhibited a similar growth pattern to the wild-type *E. coli* strain. This analysis, when combined with the macromolecular synthesis assay and proteomics results, supports the notion that thioredoxin reductase is not the sole target of auranofin in bacteria. Additionally, the outer membrane, and not the glutathione system alone, is responsible for limiting auranofin’s antibacterial activity against Gram-negative bacteria.

### Auranofin inhibits *S. aureus* toxin production

**Figure 4:**
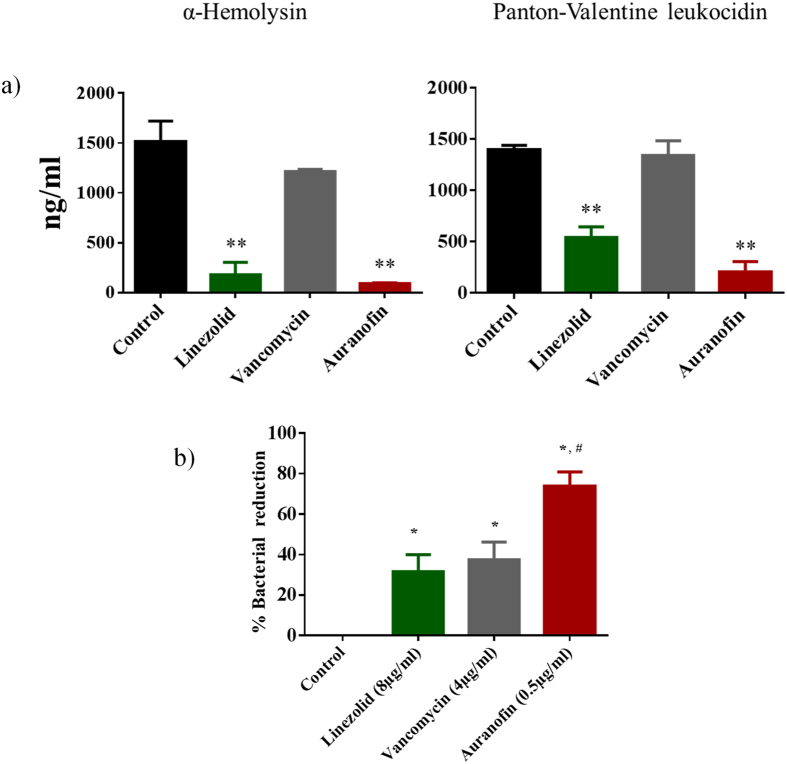
Auranofin inhibits MRSA toxin production and effectively clears intracellular bacteria. **(a)** Toxin production (ng/ml) in *S. aureus* MRSA USA300 after treatment with auranofin or control antibiotics (linezolid or vancomycin) for one hour (data corrected for organism burden). The results are presented as mean ± SD (n = 3). Statistical analysis was done by two-tailed Student’s ‘t’ test. Asterisks (**) indicate statistical significance in relation to the control (DMSO or water). *P* values of (**P ≤ 0.01) are considered significant. Detailed “P” values are listed below. α-hemolysin: control vs linezolid: 0.0027, control vs auranofin: 0.001. Panton-Valentine leukocidin: control vs linezolid: 0.0017, control vs auranofin: 0.0040. **(b)** MRSA USA300 infected J774A.1 cells were treated with auranofin and control antibiotics (vancomycin or linezolid) for 24 hours and the percent bacterial reduction was calculated compared to untreated control groups. The results are given as mean ± SD (n = 3). Two-tailed Student’s ‘t’ test was employed and *P* values of (*^,#^ ≤ 0.05) are deemed significant. Auranofin was compared to controls (*) and to antibiotics (^#^). Detailed “P” values are listed below. Control vs linezolid: 0.0234, control vs vancomycin: 0.021, control vs auranofin: 0.02031, linezolid vs auranofin: 0.0397, vancomycin vs auranofin: 0.0491.

Confirmation that auranofin inhibits bacterial protein synthesis by macromolecular synthesis assay, led us to inquire whether this drug would be capable of suppressing the production of key virulence factors, such as toxins, produced by pathogens like MRSA. Antimicrobials capable of disrupting or suppressing bacterial protein synthesis, including agents like linezolid, are valuable and preferred options for treating patients impacted by toxin-mediated bacterial infections, such as toxic shock syndrome (TSS) and pneumonia caused by *S. aureus*^23^,^24^,^25^,^26^. For example, inhibition of protein synthesis and the subsequent suppression of toxin production is one of the advantages of linezolid’s mechanism of action over vancomycin^23^,^24^,^25^,^26^. Therefore to assess the capability of auranofin to dampen production of key *S.-aureus* toxins, ELISA was utilized to detect toxin production for MRSA USA300 treated with auranofin and two control antibiotics (vancomycin and linezolid). Auranofin significantly inhibited production of two major *S. aureus* toxins including Panton-Valentine leukocidin (PVL) and α-hemolysin (Hla) (Fig. 4a). These results indicate that auranofin, similar to linezolid, possesses an advantage in the management of toxin-mediated staphylococcal infections due to its ability to suppress production of key staphylococcal toxins.

### Auranofin effectively clears intracellular bacteria

As auranofin exhibited potent anti-MRSA activity against extracellular bacteria, we were curious to explore the ability of auranofin to eliminate MRSA harboring inside eukaryotic cells. MRSA is capable of entering multiple cell types, including macrophages, in mammalian tissues thus permitting it to evade host defenses and permitting an infection to persist for an extended time period^27^. Such infections are particularly challenging to treat given many antibiotics are unable to permeate cellular membranes to gain entry into these intracellular niches to kill MRSA^28^,^29^,^30^,^31^,^32^,^33^,^34^. One such example is the antibiotic vancomycin, which has a clinical failure rate of more than 40% in treating *S. aureus* pneumonia; failure is attributed in part to the inability of vancomycin to penetrate infected alveolar macrophages to kill MRSA^35^. In order to investigate the efficacy of auranofin in clearing intracellular MRSA, this drug was tested against macrophage cells (J774.A1) infected with MRSA. At a non-toxic concentration of 0.5 μg/ml (Supplementary Figure 4); auranofin effectively clears more than 60% of intracellular MRSA (Fig. 4b). In contrast, conventional antibiotics such as linezolid (8 μg/ml) and vancomycin (4 μg/ml) are not able to reduce the bacterial burden inside infected macrophages by more than 30% (Fig. 4b). Altogether the results suggest that auranofin is capable of eradicating MRSA harboring inside mammalian cells. These findings suggest that auranofin is a potential valuable treatment option for challenging infections/diseases (such as pneumonia) where MRSA reside inside host cells.

### Auranofin rescues mice from MRSA septicemic infection

**Figure 5:**
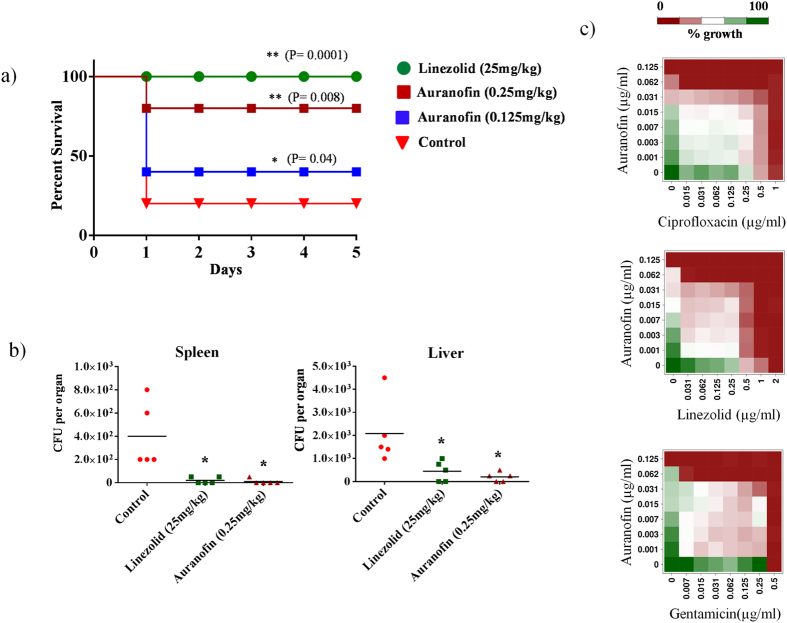
Auranofin is effective in a mouse model of MRSA septicemic infection. **(a)** Ten mice per group were infected (i.p) with lethal dose of MRSA USA300 and treated orally with auranofin (0.125 or 0.25 mg/kg), linezolid (25 mg/kg), or the vehicle alone for three days (one dose per day). Mice were monitored for five days and the percent survival was calculated. A log rank test was performed using 95% confidence intervals and the statistical significance was calculated in order to compare treated to control groups. *P* values of (* ≤ 0.05) (**P ≤ 0.01) are considered as significant. Detailed “P” values are listed below. Control vs linezolid (25 mg/kg):0.0001, Control vs auranofin (0.25 mg/kg): 0.0008, Control vs auranofin (0.125 mg/kg): 0.04. **(b)** Five mice per group were infected (i.p) with non-lethal dose of MRSA USA300 and treated orally with auranofin (0.25 mg/kg), linezolid (25 mg/kg), or the vehicle alone for two days (one dose per day). 24 hours after the last treatment, mice were euthanized and their spleen and liver were excised and homogenized in TSB to count viable MRSA colonies. The number of CFU from each mouse is plotted as individual points. Statistical analysis was conducted using the two-tailed Student’s ‘t’ test and *P* values of (* ≤ 0.05) are considered as significant. Detailed “P” values are listed below. Spleen: Control vs linezolid (25 mg/kg):0.0173, Control vs auranofin (0.25 mg/kg): 0.0153. Liver: Control vs linezolid (25 mg/kg):0.0481, Control vs auranofin (0.25 mg/kg): 0.0178. **(c)** Auranofin in combination with systemic antimicrobials effectively inhibits the growth of *S. aureus*. Growth of MRSA USA300 was measured after incubating with auranofin, control antibiotics, or a combination of auranofin + a control antibiotic. The checkerboard assay was performed by diluting one drug along the ordinate and the second drug along the abscissa of a 96-well plate. Percent bacterial growth was measured using a spectrophotometer.

The efficacy of auranofin was evaluated in both a lethal and non-lethal systemic MRSA infection model. In the lethal septicemic study, mice were infected intraperitoneally with MRSA USA300. One hour post-infection, four groups of mice (n = 10 mice per group) were treated orally with auranofin at a clinical dose of 0.125 or 0.25 mg per kg, linezolid at a dose of 25 mg per kg, or the vehicle alone as a control. Mice were treated once daily for three days and monitored for a total of five days. Both auranofin and linezolid provided a significant protection from mortality (Fig. 5a). The survival rate of infected mice improved dramatically when the dose of auranofin was increased. 80% of mice that received a higher dose of auranofin, (0.25 mg per kg) survived for five days. All mice in the group that received linezolid (25 mg per kg) survived for five days. These results suggest that the potent *in vitro* activity of auranofin translates *in vivo* in protecting mice from septicemic MRSA infection.

Next we moved to study the efficacy of auranofin in reducing the burden of MRSA in a non-lethal septicemic mouse model. Mice were infected with a non-lethal dose of MRSA USA300 and each group of mice received two oral doses of auranofin (0.25 mg per kg), linezolid (25 mg per kg) or the vehicle alone. As depicted in Fig. 5b, auranofin and linezolid produced a significant reduction in mean bacterial load in murine organs including the spleen and liver. Both treatment with auranofin and treatment with linezolid reduced the mean bacterial load by more than 95% in the spleen (Fig. 5b). However, in the liver, auranofin produced a 90% reduction in MRSA load whereas linezolid was only able to reduce the burden of MRSA by 70% (Fig. 5b).

### Combination therapy of auranofin with systemic antimicrobials

Utilizing a single agent to treat bacterial infections in the clinical setting appears to have become less effective with the rise of additional strains of multidrug-resistant *S. aureus*^36^,^37^. Combining two or more antibiotics together for the treatment of MRSA infections has been explored as an alternative strategy in the healthcare setting in order to improve the morbidity associated with these infections and to reduce the potential emergence of additional resistant strains^36^,^38^,^39^. Therefore, we investigated auranofin’s ability to be used in combination with antimicrobials frequently used to treat systemic MRSA infections. When tested against a highly-prevalent strain of MRSA USA300, auranofin exhibited an additive effect in inhibiting bacterial growth when combined with the antibiotics ciprofloxacin, linezolid and gentamicin (average fractional inhibitory concentration, FIC index = 0.5 to 1) (Fig. 5c). Thus the above results indicate auranofin is a potential candidate for further investigation as a partner with conventional antimicrobials for the treatment of systemic staphylococcal infections.

## Discussion

Methicillin-resistant *Staphylococcus aureus* infections continue to pose a significant challenge to healthcare providers in part due to the diminishing arsenal of effective antibiotics available to treat infected patients. The development of novel antibacterial treatments utilizing the traditional approach in drug discovery has not kept pace with the rapid emergence of bacterial resistance to conventional antibiotics. This has led researchers to explore alternative methods to discover new treatment options for bacterial infections; one method that is less time-consuming and more financially viable is repurposing drugs (initially approved for other clinical indications) that possess potent antimicrobial activity. Auranofin is an example of a clinical drug that has been successfully repurposed recently for another indication. Initially approved as a treatment option for patients suffering from rheumatoid arthritis, auranofin was granted orphan-drug status from the FDA as an anti-parasitic agent intended for treatment of human amebiasis in 2012^11^.

The successful repurposing of auranofin as an anti-parasitic agent paved the way for researchers to explore other clinical applications for auranofin. Recent studies, including the present work, demonstrate that auranofin possesses potent antibacterial activity against important Gram-positive pathogens, including MRSA. One of the key structural features of auranofin is that it is an organogold compound; however unlike other gold compounds including sodium aurothiomalate and sodium aurothioglucose hydrate (MIC >16 μg/ml), auranofin exhibits potent antibacterial activity against an array of different Gram-positive bacteria (including *S. aureus*, *E. faecium*, *E. feacalis*, *S. pneumoniae and S. agalactiae*) with an average minimum inhibitory concentration (0.125 μg/ml) eighteen times lower than the achievable drug concentration in human plasma (2.37 μg/ml which is equivalent to a mean steady-state blood gold concentration of 3.5 μM)^11^. This is in agreement with previous published studies^12^,^14^; however several of these reports have indicated that auranofin lacks antibacterial activity against Gram-negative bacteria. A recent study suggested that this lack of activity was due to the presence of the glutathione system in Gram-negative bacteria which helps to mediate resistance to auranofin in these pathogens^13^. However, when we assessed auranofin’s antibacterial activity against both wild-type and Origami-2 (*trxb*/*gor* double mutant) *E. coli* mutant strains, neither strain was susceptible to auranofin even at a concentration of 16 μg/ml^40^. This suggests an alternative mechanism may be responsible for the lack of activity observed with auranofin against Gram-negative bacteria.

Further investigation revealed that the presence of the outer membrane in Gram-negative bacteria, and not the glutathione system, is the main culprit responsible for the lack of antibacterial activity observed. When wild-type and Origami-2 *E. coli* strains were incubated with auranofin supplemented with a subinhibitory concentration of PMBN (to permeabilize the outer membrane), both strains showed similar sensitivity to auranofin with a MIC value of 0.5 μg/ml (Table 2). This observation was further validated by assessing the growth of wild-type and double mutant *E. coli* strains in the presence of increasing concentrations of auranofin (with or without PBMN) (Fig. 3). Once again, the viability of the Origami-2 double mutant was severely impacted by the presence of auranofin (in the presence of a subinhibitory concentration of PBMN); however, in the absence of PBMN, the double mutant strain exhibited a similar growth pattern to the wild-type *E. coli* strain. Thus the lack of direct antibacterial activity of auranofin observed against Gram-negative bacteria appears to be a byproduct of the barrier imposed by the outer membrane in addition to the presence of active efflux pumps more so than the presence of the glutathione system.

Confirmation of auranofin’s potent antibacterial activity led us to next explore the potential mechanism of action (MOA) against *S. aureus*. Previous studies have found that auranofin inhibits *Clostridium difficile* and *Treponema denticola* growth through the disruption of selenium metabolism^41^,^42^. We hypothesized that the MOA of auranofin in *S. aureus* differs from the MOA in *C. difficile* and *T. denticola* due to the absence of selenoproteins in *S. aureus*^43^. In order to examine this hypothesis, we tested the activity of auranofin on *S. aureus* cultures supplemented with selenium in the form of selenite or L-selenocysteine^41^,^42^. Unlike in *C. difficile* and *T. denticola*, our selenium supplementation did not reverse the inhibitory action of auranofin observed with *S. aureus* (data not shown). This clearly indicates that the MOA of auranofin differs between *S. aureus* and *C. difficile*. Next, we attempted to generate a *S. aureus* mutant that is resistant to auranofin. Determination of mutation frequencies for resistance to auranofin were carried out as described before^44^. No colonies resistant to auranofin at three-, five-, or ten-fold the MIC were detected which is in agreement with a previous report^13^.

The inability to generate a resistant mutant to auranofin suggests this drug may have multiple targets or possess a nonspecific mode of action against *S. aureus*^45^. To assess this, a macromolecular synthesis assay was employed testing auranofin at different concentrations against *S. aureus*. Interestingly, at a subinhibitory concentration (0.5 × MIC), auranofin leads to significant reduction in both the cell wall and DNA biosynthetic pathways. At its MIC, auranofin also suppresses bacterial protein synthesis, indicating auranofin may in fact have a complex mode of action against *S. aureus*. Harbut *et al.*’s recently reported auranofin exerts its antibacterial activity primarily by targeting thiol-redox homeostasis through direct inhibition of the thioredoxin reductase enzyme (TrxB in *Staphylococcus aureus* and TrxB2 in *Mycobacterium tuberculosis*). While inhibition of TrxB activity in *S. aureus* can lead to inhibition of DNA synthesis, it does not explain the inhibition of cell wall synthesis observed with auranofin. Taken altogether, our analysis indicates that the thioredoxin reductase enzyme most likely is not the sole target of auranofin in *S. aureus* and in Gram-negative bacteria; this is in agreement with a recent report investigating auranofin’s antibacterial activity against *Streptococcus pneumoniae* and *S. aureus*^15^. Further studies are needed to fully elucidate the exact antibacterial molecular target(s) of auranofin.

In the course of investigating auranofin’s mode of action via macromolecular synthesis, we discovered that auranofin inhibits protein synthesis in *S. aureus*. This discovery led us to analyze whether auranofin’s inhibitory activity against bacterial protein synthesis would lead to suppression in the production of key toxins in *S. aureus*. Our study revealed that auranofin is capable of inhibiting production of both Panton-Valentine leukocidin and α-hemolysin, two pore-forming cytotoxins that injure host immune cells and promote infection^46^. Thus, in addition to its direct –cidal effect on bacteria, auranofin may alleviate the morbidity associated with MRSA infections by limiting bacteria from generating harmful toxins.

We next moved to confirm auranofin’s antibacterial ability *in vivo* using two murine MRSA systemic infection models (non-lethal and lethal). Both *in vivo* studies performed in mice confirmed auranofin retains its antibacterial activity *in vivo*. In addition to this, auranofin demonstrated the ability to eradicate intracellular MRSA present inside infected macrophage cells; this expands the potential application of auranofin for use in treatment of systemic MRSA infections. Furthermore, auranofin demonstrated additive activity when combined with antibiotics traditionally used to treat systemic MRSA infections which is in agreement with previous a study^13^. Thus, auranofin has potential use both as a single agent and as a combinatorial partner with conventional antibiotics to treat MRSA infections. This latter statement is important given the emergence of resistance to systemic antimicrobials currently used in the clinic; pairing these antibiotics with auranofin may stymie the rate at which resistance to these antibiotics arises. Finally, because of increased interest in repurposing auranofin, a Phase II clinical trial seeking to determine the pharmacokinetic parameters and the safety of increased doses of auranofin are currently underway (ClinicalTrials.gov identifier: NCT01419691 and NCT02089048). This strongly supports the postulate that auranofin has considerable promise to be repurposed as an antibacterial agent for the treatment of systemic bacterial infections.

## Methods

### Bacterial strains and reagents

Bacterial strains used in this study are presented in Tables 1 and 2. Mueller-Hinton broth (MHB) was purchased from Sigma-Aldrich while Trypticase soy broth (TSB), Trypticase soy agar (TSA), and mannitol salt agar (MSA) were purchased from Becton, Dickinson and Company (Cockeysville, MD). Auranofin (Enzo Life Sciences), vancomycin hydrochloride (Gold Biotechnology) and linezolid (Selleck Chemicals) were all purchased from commercial vendors.

### Antibacterial assays

The broth microdilution method was employed to determine the MICs of all test agents (tested in triplicate) as per the Clinical and Laboratory Standards Institute (CLSI) guidelines^47^. Test agents were incubated with bacteria for 16 hours at 37 °C prior to determining the MIC. The MIC was classified as the lowest concentration of drug capable of inhibiting visible growth of bacteria by visual inspection.

### Gram-negative bacteria outer membrane permeabilization assay

The MIC of auranofin and control antibiotics, in the presence of polymixin B nonapeptide (PMBN), against Gram-negative bacteria was measured as described in the antibacterial assay section above. A subinhibitory concentration of PMBN (10 μg/ml) was added to TSB to increase the outer membrane permeability and facilitate the entrance of auranofin, as described elsewhere^17^,^18^.

### Macromolecular synthesis assay

*S. aureus* ATCC 29213 was used for the macromolecular synthesis assay and the assay was carried out using auranofin and control antibiotics (ciprofloxacin, rifampicin, linezolid, vancomycin and cerulenin) as described elsewhere^9^,^48^.

### Proteomics analysis

#### Sample Preparation

An overnight culture of MRSA USA300 cells were treated with 10 × MIC of auranofin (1.25 μg/ml), linezolid (20 μg/ml) and vancomycin (10 μg/ml) for one hour at 37 °C. Bacterial cells were centrifuged and sequence grade Lys-C/Trypsin (Promega) was used to enzymatically digest samples. Samples were reduced and alkylated prior to digestion. All trypsin digestions were carried out in a Barocycler NEP2320 (PBI) at 50 °C under 20 kpsi for two hours. After digestion, samples were cleaned using MicroSpin C18 columns (Nest Group, Inc.) and the resulting pellets were re-suspended in 97% H_2_O/3% ACN/0.1% FA. A small aliquot (5 μL) of sample was analyzed via nanoLC-MS/MS^49^.

#### LC-MS/MS

Samples were run on an Eksigent 425 nanoLC system coupled to the Triple TOF 5600 plus^50^. The gradient was 120 min at 300 nl/min over the cHiPLC–nanoflex system. The trap column was a Nano cHiPLC 200 μm × 0.5 mm ChromXP C18-CL 3 μm 120 Å followed by the analytical column, the Nano cHiPLC 75 μm × 15 cm ChromXP C18-CL 3 μm 120 Å. The sample was injected into the Triple TOF 5600 plus through the Nanospray III source. Data acquisition was performed at 50 precursors at 50 min/scan.

#### Analysis

WIFF files from mass spectrometric analysis were processed using the MaxQuant computational proteomics platform version 1.5.2.8^51^. The peak list generated was screened against the *Bos taurus* (41521 entries unreviewed) and *Staphylococcus aureus* (10972 entries reviewed) sequence from UNIPROT retrieved on 04/10/2015 and a common contaminants database. The following settings were used for MaxQuant: initial precursor and fragment mass tolerance set to 0.07 and 0.02 Da respectively, a minimum peptides length of seven amino acids, data was analyzed with ‘Label-free quantification’ (LFQ) checked and the ‘Match between runs’ interval set to 1 min, the FASTA databases were randomized and the protein FDR was set to 5%, enzyme trypsin permitted two missed cleavage and three modifications per peptide, fixed modifications were carbamidomethyl (C), variable modifications were set to Acetyl (Protein N-term) and Oxidation (M). The MaxQuant results used in-house script, and the average LFQ intensity values for the technical replicates were used for each sample. Both the *Bos taurus* and the common contaminant proteins were removed. Values were transformed [log_2_(x)] and the missing values were inputted using the average values of all samples. The heat maps and statistical analyses were performed in the R environment (www.cran.r-project.org) and Qlucore OMICS explorer (version 3.0, Qlucore, Lund, Sweden). A one-way analysis of variance (ANOVA) was performed on the LFQ intensities and only proteins with *P* < 0.05 were selected for further analyses.

### Growth curve of *E. coli* in the presence of auranofin

Wild-type and *trxB*/*gor* double mutant *E. coli* strains (wild-type: novablue (DE3)-K12, *trxB/gor* double mutant: Origami-2) were incubated with indicated concentration of auranofin in the presence and absence of PMBN (10 μg/ml) for 16 hours at 37 °C. Bacterial growth was monitored using a spectrophotometer (OD = 600 nm).

### Analysis of *S. aureus* toxin production by ELISA

The effect of auranofin and two control antibiotics (vancomycin and linezolid) on production of two key *S. aureus* toxins (α-hemolysin and Panton-Valentine leukocidin) was measured by ELISA as has been previously described^9^.

### Intracellular infection assay

J774A.1 murine macrophage-like cells were infected with MRSA USA300 for 30 min at a multiplicity of infection (MOI) ratio of 1:100. Infected cells were subsequently washed three times with DMEM medium containing 10 IU lysostaphin^52^. Auranofin (0.5 μg/ml), vancomycin (4 μg/ml) and linezolid (8 μg/ml), in triplicates, in complete DMEM medium containing 4 IU lysostaphin was then added. After 24 hours of incubation at 37 °C (with 5% CO_2_), the cells were washed three times with phosphate-buffered saline (PBS) and lysed with 0.1% Triton X-100 (Sigma-Aldrich). Cell lysates were plated onto TSA plates and MRSA colony forming units (CFU) were counted after incubation of plates for 24 hours at 37 °C.

### Mice studies

Eight week old female BALB/c mice (Harlan Laboratories, Indianapolis, IN) were used in all mice studies. The animal care and all experiments were approved and performed in accordance with the guidelines approved by Purdue University Animal Care and Use Committee (PACUC). Eight-week old female BALB/c mice (n = 10 per group) were used and the study was carried out as described before^53^.

### Systemic - lethal infection

An overnight culture of MRSA USA300 cells were washed and re-suspended in PBS. Each mouse received an intraperitoneal (i.p.) injection (200 μl) containing the bacterial suspension (9 × 10^9^ CFU). One hour after infection, mice were divided into four groups (ten mice per group). Mice were treated orally with auranofin (either 0.125 or 0.25 mg/kg), linezolid (25 mg/kg), or the vehicle alone (10% ethanol). Treatment was provided once daily for three days following infection. Mortality was monitored daily for five days and the moribund mice were euthanized humanely using CO_2_ asphyxiation.

### Systemic–non-lethal infection

The infection protocol was carried out as described above (systemic-lethal infection) with the following exceptions. Each mouse received an i.p. injection containing 2 × 10^7^ CFU MRSA USA300. Mice were divided into three groups (five mice per group) and treated orally with auranofin (0.25 mg/kg), linezolid (25 mg/kg), or vehicle (10% ethanol) alone. Mice were treated once daily for two days. Twenty-four hours after the last dose, mice were euthanized and their spleen and liver were excised, homogenized in TSB, plated onto MSA plates, and incubated at 37 °C for 24 hours prior to counting MRSA CFU post-treatment.

### Combination testing of auranofin with commercial antibiotics

Additive activity of auranofin with conventional antibiotics (ciprofloxacin, linezolid and gentamicin) was evaluated as described in a previous study^54^,^55^,^56^,^57^,^58^. Briefly, MRSA USA300 was incubated with auranofin, control antibiotics, or a combination of auranofin + a control antibiotic at different concentrations for 16 hours. Next, the optical density (at 600 nm) was measured using a spectrophotometer. Percent bacterial growth for each treatment regimen was calculated and presented.

### Statistical analyses

Statistical analyses were assessed using GraphPad Prism 6.0 (Graph Pad Software, La Jolla, CA). *P* values were calculated via the Student *t* test or Kaplan-Meier (log rank) survival test as indicated. *P* values of ≤0.05 were deemed significant.

## Additional Information

**How to cite this article**: Thangamani, S. *et al.* Antibacterial activity and mechanism of action of auranofin against multi-drug resistant bacterial pathogens. *Sci. Rep.* **6**, 22571; doi: 10.1038/srep22571 (2016).

## Supplementary Material

Supplementary Information

## Figures and Tables

**Table 1:** MICs of auranofin and control antibiotics against Gram-positive bacteria.

Strain ID	Source	Phenotypic Characteristics	Auranofin MIC (μg/ml)	Linezolid MIC (μg/ml)	Vancomycin MIC (μg/ml)
MRSA (USA100)	United States (Ohio)	Resistant to ciprofloxacin, clindamycin,	0.125	2	2
		erythromycin			
MRSA (USA200)	United States (North Carolina)	Resistant to clindamycin, methicillin	0.0625	2	1
		erythromycin, gentamicin,			
MRSA (USA300)	United States (Mississippi)	Resistant to erythromycin, methicillin,	0.125	2	1
		tetracycline			
MRSA (USA400)	United States (North Dakota)	Resistant to methicillin, tetracycline	0.0625	2	1
MRSA (USA700)	United States (Louisiana)	Resistant to erythromycin, methicillin	0.125	4	1
MRSA (USA800)	United States (Washington)	Resistant to methicillin	0.0625	4	1
MRSA (USA1000)	United States (Vermont)	Resistant to erythromycin, methicillin	0.125	2	1
MRSA (USA1100)	United States (Alaska)	Resistant to methicillin	0.125	2	1
*E. faecalis* ATCC49533	Blood, Wisconsin	Resistant to streptomycin	0.125	2	1
*E. faecalis* ATCC7080	Meat involved in food poisoning, New York	–	0.125	2	1
*E. faecalis* ATCC 51229 (VRE)	Peritoneal fluid, St. Louis, MO	Resistant to Vancomycin. Sensitive to Teicoplanin	0.125	2	8
*E. faecium* E0120 (VRE)	Ascites fluid, Netherlands	Resistant to gentamicin and vancomycin	0.25	2	>128
*E. faecium* ATCC6569	Human feces	–	0.125	2	1
*S. pneumoniae* 51916	Human CSF, USA	Resistant to cephalosporins	0.25	1	1
*S. pneumoniae* 70677	Human patient, Czechoslovakia	Resistant to erythromycin, penicillin, and tetracycline	0.25	1	1
*Streptococcus agalactiae* MNZ938	Human blood	Beta-hemolytic, Serogroup: Group B	0.0625	0.25	0.25
*Streptococcus agalactiae* MNZ 933	Human blood	Beta-hemolytic, Serogroup: Group B	0.0625	0.25	0.5
*Streptococcus agalactiae* MNZ 929	Human blood	Beta-hemolytic, Serogroup: Group B	0.0015	0.25	0.25

**Table 2:** MICs of auranofin and control antibiotics against Gram-negative bacteria.

Bacteria	Minimum Inhibitory Concentration (MIC) (μg/ml)										
	PMBN	Auranofin		Erythromycin		Fusidic acid		Linezolid		Daptomycin	
		PMBN		PMBN		PMBN		PMBN		PMBN	
		(−)	(+)	(−)	(+)	(−)	(+)	(−)	(+)	(−)	(+)
*Acinetobacter baumannii* ATCC BAA19606	>256	16	0.25	64	0.5	64	0.5	256	64	>256	>256
*Acinetobacter baumannii* ATCC BAA1605	>256	16	0.5	64	0.5	128	1	>256	128	>256	>256
*Acinetobacter baumannii* ATCC BAA747	>256	16	0.25	64	1	128	0.5	>256	64	>256	>256
*Escherichia coli* O157:H7 ATCC 700728	256	64	0.5	128	1	>256	16	256	16	>256	>256
*Escherichia coli* O157:H7 ATCC 35150	256	32	0.5	128	1	>256	16	>256	16	>256	>256
*Salmonella Typhimurium* ATCC 700720	>256	128	1	256	2	>256	16	256	16	>256	>256
*Klebsiella pneumoniae* ATCC BAA 2146	>256	256	0.5	>256	128	>256	32	>256	64	>256	>256
*Klebsiella pneumoniae* ATCC BAA 1705	>256	256	1	>256	64	>256	64	>256	128	>256	>256
*Pseudomonas aeruginosa* ATCC 9721	>256	>256	0.25	>256	1	>256	1	>256	4	>256	>256
*Pseudomonas aeruginosa* ATCC 27853	>256	256	0.125	256	1	>256	1	>256	16	>256	>256
*Pseudomonas aeruginosa* ATCC BAA-1744	>256	>256	0.25	>256	1	>256	1	>256	16	>256	>256
*Pseudomonas aeruginosa* ATCC 25619	>256	256	0.25	256	1	>256	1	>256	8	>256	>256
*Pseudomonas aeruginosa* ATCC 35032	>256	>256	0.5	>256	1	>256	1	>256	8	>256	>256
*Pseudomonas aeruginosa* ATCC 10145	>256	256	0.25	256	1	>256	2	>256	8	>256	>256
*Pseudomonas aeruginosa* ATCC 15442	>256	>256	0.25	>256	2	>256	1	>256	16	>256	>256
*Escherichia coli* 1411	>256	32	0.5	32	4	>256	4	>256	16	>256	>256
*Escherichia coli* SM1411*∆ acrAB*	>256	8	0.5	0.03	<0.03	8	<0.03	32	2	>256	>256
*Escherichia coli* (Novablue (DE3)-K12)	256	16	0.5	16	0.5	>256	0.5	>256	16	>256	>256
*Escherichia coli* (Origami-2) (*trxB/gor* mutant)	256	16	0.5	32	0.5	256	0.06	128	16	>256	>256

PMBN polymyxin B nonapeptide: (−) No PMBN was added to the media; (+) (10 μg/ml) of PMBN was added to the media.
