# Supplementary material for: Antibacterial activity and mechanism of action of auranofin against multi-drug resistant bacterial pathogens
Source: Sci Rep. 2016 Mar 3;6:22571. doi: 10.1038/srep22571 (PMC4776257; doi:10.1038/srep22571)
Supplement: Supplementary Information [file srep22571-s1.pdf]

SUPPLEMENTARY MATERIALS

Antibacterial activity and mechanism of action of auranofin against multi-drug resistant bacterial pathogens

Shankar Thangamani[1], Haroon Mohammad[1], Mostafa F.N. Abushahba[1,2], Tiago J.P. Sobreira[3], Victoria E. Hedrick[3], Lake N. Paul[3] and Mohamed N. Seleem[1]*

[1]Department of Comparative Pathobiology, Purdue University College of Veterinary Medicine, West Lafayette, IN, USA

[2]Faculty of Veterinary Medicine, Assiut University, Assiut, Egypt.

[3]Bindley Bioscience Center, Purdue University, West Lafayette, IN, USA

***Corresponding author: Mohamed N. Seleem. Email: mseleem@purdue.edu

MATERIALS AND METHODS

Growth curve of *S. aureus* in the presence of auranofin: MRSA USA300 was incubated with indicated concentration of auranofin for 16 hours at 37°C and the percent bacterial growth was monitored using a spectrophotometer (OD = 600 nm).

Time kill assay: An overnight culture of MRSA USA300 was diluted to 6×10^5 CFU/mL and treated with 5 × MIC of auranofin, vancomycin or linezolid (in triplicate) in Mueller-Hinton broth and incubated at 37°C. Samples were collected at indicated time points, serially diluted in PBS, and transferred to TSA plates. Plates were incubated at 37°C for 24 hours prior to counting MRSA colony forming units (CFU).

DNA intercalation assay: DNA intercalation assay was carried out using pUC 18 plasmid as described elsewhere (*1*). Briefly, 250 ng of plasmid DNA was incubated with the indicated concentration of auranofin and doxorubicin in a total volume of 25 µl and the reaction mixtures were incubated for 30 min at 37°C. An electrophoretic assay was run using 1% agarose gel without ethidium bromide at 50 volts for 4 hours. The gel was stained with ethidium bromide and visualized for DNA mobility.

Cytotoxicity assay: *In vitro* toxicity assay was carried out in murine macrophage (J774A.1) cells utilizing the MTS assay as described before (*2*). Briefly, auranofin at a concentration ranging from 0 to 256 µg/ml was added to cells in a 96-well tissue culture plate. After 24 hours of incubation, the cytotoxicity effect of auranofin was measured by the addition of MTS assay reagent 3-(4,5-dimethylthiazol-2-yl)-5-(3-carboxymethoxyphenyl)-2-(4-sulfophenyl)-2H tetrazolium). Results are expressed as percent viability of auranofin-treated cells in comparison to cells treated with DMSO.

SUPPLEMENTARY FIGURES:

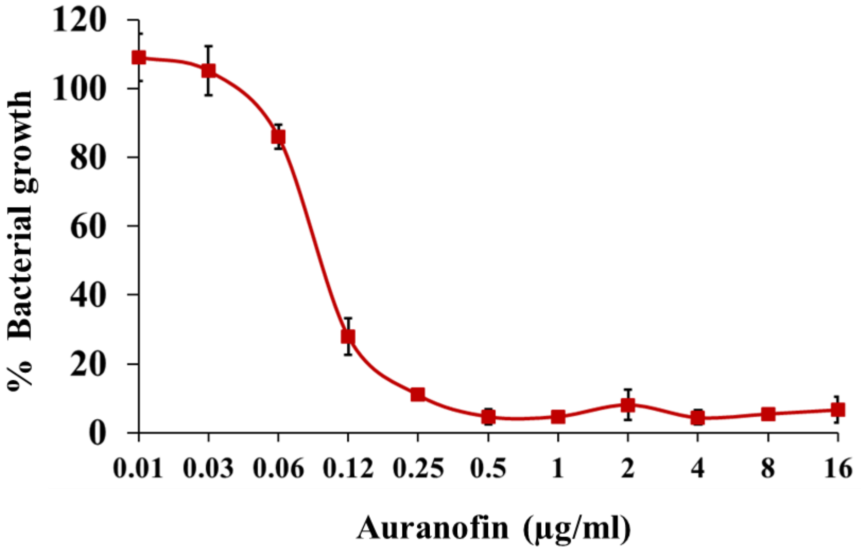

Fig. S1. Growth curve of MRSA USA300 in the presence of auranofin. Bacteria were incubated with indicated concentrations of auranofin and the growth was measured using a spectrophotometer.

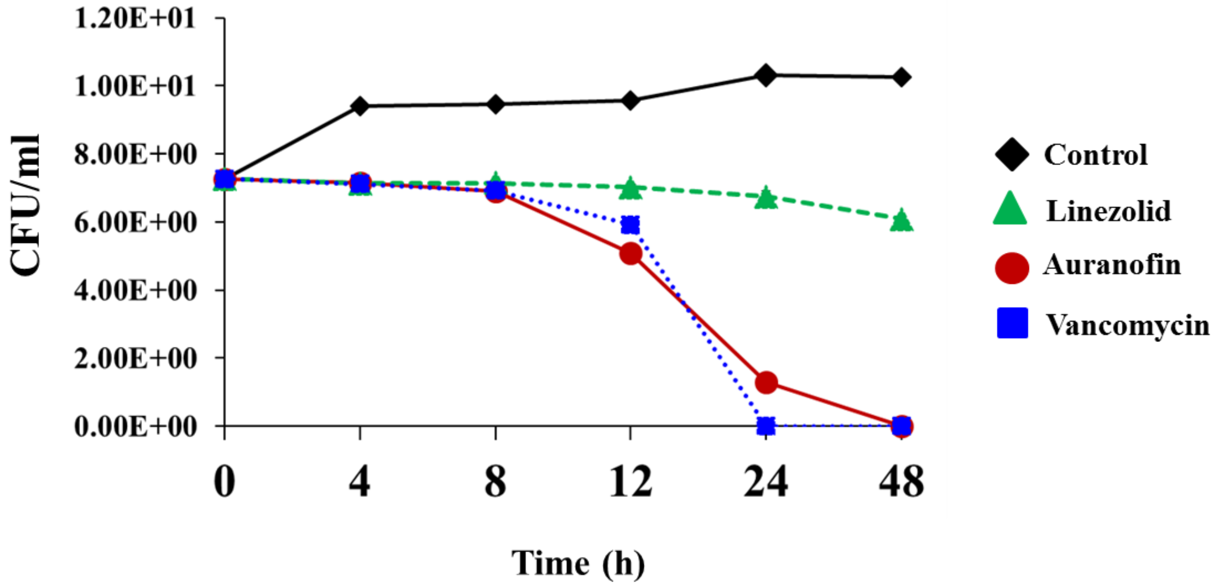

Fig. S2. Time-kill assay for auranofin tested against *S. aureus*. Killing kinetics of auranofin and antibiotics (vancomycin and linezolid) at 5 × MIC against MRSA USA300 in MHB are displayed. The results are presented as mean ± SD (n = 3). Data without error bars indicate that the SD is too small to be seen.

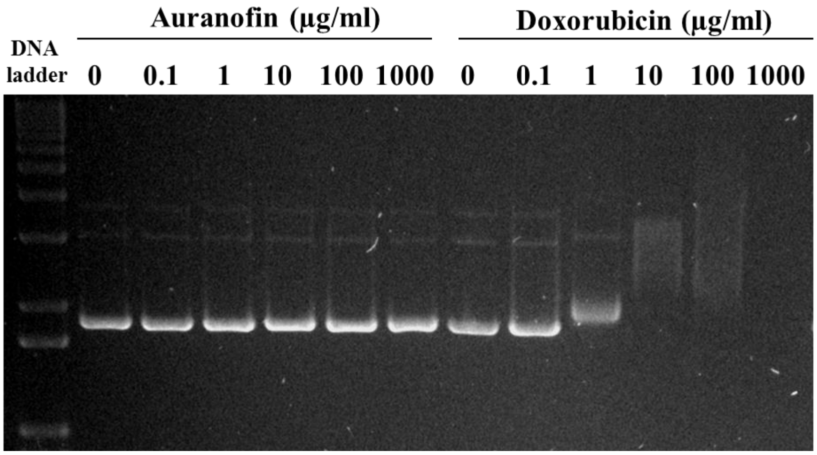

Fig. S3. DNA mobility assay in the presence of auranofin and doxorubicin. pUC 18 plasmid was incubated with the indicated concentration of auranofin and doxorubicin for 30 min at 37°C. An electrophoretic assay was run using 1% agarose gel, stained with ethidium bromide and visualized for DNA mobility.

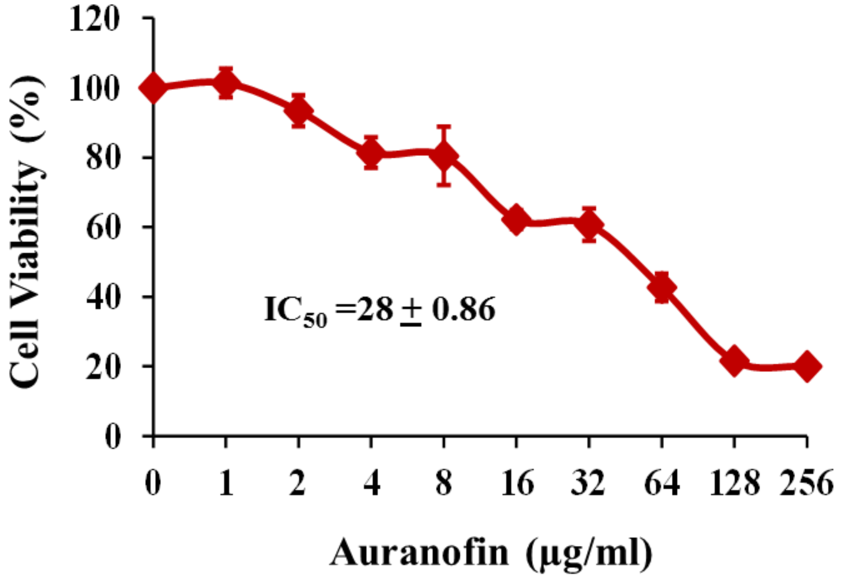

Fig. S4. Cytotoxicity assay in murine macrophage-like cells (J774A.1) cells. J774A.1 cells were treated with different concentration of auranofin ranging from 0 to 256µg/ml. DMSO was used as a negative control. Cell viability was measured by MTS assay and IC_{50} of auranofin to cause cytotoxicity in J774A.1 cells was calculated.

References:

1. Y. S. Wu, K. R. Koch, V. R. Abratt, H. H. Klump, Intercalation into the DNA double helix and in vivo biological activity of water-soluble planar [Pt(diimine)(N,N-dihydroxyethyl-N'-benzoylthioureato)]+Cl- complexes: a study of their thermal stability, their CD spectra and their gel mobility. *Archives of biochemistry and biophysics* **440**, 28-37 (2005).
2. S. Thangamani, W. Younis, M. N. Seleem, Repurposing Clinical Molecule Ebselen to Combat Drug Resistant Pathogens. *PloS one* **10**, e0133877 (2015).
